# The enhancement of teacher immunity in a professional development course: Contributions from supervisory observation and the observer

**DOI:** 10.1016/j.heliyon.2024.e39355

**Published:** 2024-10-21

**Authors:** Mohammad Sheikhi, Parviz Alavinia

**Affiliations:** Department of English Language and Literature, Urmia University, Urmia, Iran

**Keywords:** Language teacher immunity, Classroom observation, Self-organization, Professional development

## Abstract

Classroom observation has been reported to be stressful for language teachers since it can disrupt the equilibrium and push the teachers towards maladaptive language teacher immunity. The present study is an attempt to shed light on the way classroom observation and a professional development (PD) course on classroom observation contribute to language teacher immunity development through self-organization lens. To this end, the researchers in the current study designed an explanatory sequential mixed method study and 13 novice language teachers were invited to fill language teacher immunity scale before and after a professional development course. The qualitative data were collected through narrative frames and semi-structured interviews. The findings revealed that the immunity level of the students differed significantly before and after the intervention. The findings accentuate the importance of in-service programs in reforming the teachers’ narratives of classroom observations and enabling them to transform classroom observations from threats to opportunities.

## 1 Introduction

Teachers are commonly known as the “critical pillars” ([1], p. 1) of the students’ success in learning a second language. Leiter and Maslach [2] stated that teachers are the most valuable parts of every educational system, and therefore their wellbeing must be of utmost importance. Also, as the teachers are at the heart of classroom life, the atmosphere of the class is determined, to a large extent, by their thoughts, feelings, and goals [3]. Mercer [4] believes that teacher psychology research has not been researched adequately in comparison to the research on psychological traits of students.

There are a number of studies which verify that when the teachers are in a positive state of mind, they not only gain more satisfaction from teaching, but also become more creative in their job and show enhanced pedagogical skills [[5], [6], [7]]. However, language teachers come across many disturbances in their profession which culminates in the emergence of language teacher immunity [8,9]. Teacher immunity is a response in the psychology of language teachers to the adversities inherent in the teaching profession and its development can have significant influence on the teachers’ cognition and classroom practice [9,10].

Amidst the attempts targeted at boosting teachers' professional development (PD), supervisory observation seems to play the most prominent role in helping the teachers move in the right teaching trajectory to become successful teachers. However, as research has revealed, language teacher observation is among the distressing factors that disrupt the professional equilibrium of the language teachers [[11], [12], [13]]. In other words, though supervisory observation is intended to work as a cogent means for fostering teachers’ professional development, at times it is found to have counter-productive results, and lead to the emergence of language teacher immunity as a defensive mechanism. Second language teacher observations refer to the systematic process of watching, recording, and analyzing various aspects of second language (L2) teaching and learning environment [[14], [15], [16]]. These observations can be used for a variety of purposes, such as teacher training, research, or evaluation [15].

Despite the prominence of classroom observation in regulating and directing the teaching process, it seems that very scant heed has been given to the impact of supervisory teacher observation on language teacher immunity trajectories and the way intervention programs contribute to the development of immunity. Hence, aimed at bridging this gap, the researchers in the present study strove to give out a detailed image of the manner in which supervisory observation impacts different levels of immunity through the lens of self-organization. In so doing, the role a professional development course held by the observer can play in (re)shaping different subcategories of language teacher immunity was also explored.

## 2 Literature review

### 2.1 Language teacher immunity

Hiver and Dörnyei [10] introduced the concept of language teacher immunity as an armoring system which is formed in language teachers as a response to the threats embedded in a job to assist teachers to maintain their professional equilibrium [9]. The conceptualization of teacher immunity as parallel to biological immunity becomes meaningful in that it serves a protective function in the face of perturbances and complexities of L2 instructional environments. Language teacher immunity, drawing parallels from biological immunity, serves as a metaphor for the intricate process by which language instructors effectively manage and overcome the myriad challenges that permeate their profession [10].

Teacher immunity encapsulates a dynamic duality, characterized by both positive and negative manifestations. Positive language teacher immunity manifests a robust shield, enabling teachers to maintain professional equilibrium amidst the complexities of teaching, fostering resilience and effective instruction. In contrast, maladaptive language teacher immunity emerges as a maladaptive coping mechanism, potentially hindering professional growth and leading to burnout or disengagement [9,17]. Language teacher immunity, analogous to its biological counterpart develops either productively or maladaptively. Productive immunity equips teachers with resilience, enthusiasm and hope and provides them with a plethora of coping strategies to manage the inconveniences. The productively immunized teacher creates an environment that is conducive to language development and growth, rather than defensive teaching and self-preservation [[18], [19], [20], [21]]. Maladaptive immunity, on the other hand, promotes extreme risk-avoidance and aversion to change, general apathy and indifference, resignedness, callousness, and cynicism [18,19,21]. Hiver [9] delineates maladaptive immunity as a “leading factor which inhibits teacher change and growth and contributes to the unparalleled conservatism and rigidity in the language teaching profession” (p. 683).

The framework of complexity theory (CT) is a great aid in theorizing the forces of change either in society or individuals. Therefore, the developmental blueprint of immunity is informed by ‘self-organization’ and ‘emergence’ that are among the primary principles of CT. Self-organization is “the spontaneous process by which higher-level order emerges from the local interaction of disordered components.” Emergence, on the other hand, is defined as “the rise of salient, novel outcomes or configurations that are coherent at a macro level” ([10], p. 7).

Dynamic systems exhibit a remarkable ability to adapt and evolve through self-organization, a process that enables them to spontaneously restructure their internal components in response to external disruptions. External disturbances, such as new challenges or feedback, can disrupt the existing equilibrium of these systems, prompting a four-stage process of self-organization and emergence namely triggering, coupling, re-alignment, and stabilization [8,22]. This process of self-organization and emergence serves as a mechanism for dynamic systems to learn and grow from challenges, embodying the concept of “motivation-sustaining teacher immunity” in education, where teachers adapt and improve in response to pedagogical demands [22,23]. Complexity theory provides a valuable framework for understanding the multifaceted and dynamic nature of language teacher resilience, emphasizing the interplay of individual experiences, attitudes, and coping strategies. This approach acknowledges the diversity of language teacher experiences and the developmental nature of immunity.

Teacher immunity has been studied in relation to other psychological concepts and traits of language teachers, e.g. reflective teaching, emotion regulation, and immunity [24], L2 grit, and work engagement [25], wellbeing and mindfulness [26] work engagement, and psychological well-being [27] as well as engagement, emotions, and autonomy [25]. Many of the variables in these studies are categorized within the domain of positive psychology.

Haseli Songhori et al. [23], among others, intended to unveil the dominant immunity type among Iranian EFL teachers, and sought to explore how they happened to develop this type of immunity. To this end, they designed a mixed-methods study in which they distributed a questionnaire designed by Hiver [9] to identify the dominant immunity type. For the qualitative part of the study, they interviewed 13 experienced teachers to find out the ‘immunity pathways’ of the teachers. The analysis of the data revealed that maladaptive immunity was the dominant immunity type among Iranian language teachers. They stated that language teacher immunity is a continuum whose extremes are maladaptive immunity and productive immunity. Therefore, they concluded that teacher immunity is not a static and stable feature, but rather a dynamic state; however, as they claimed, the invariable presence of the disturbances caused excessive stability of maladaptive immunity among Iranian teachers.

In another study, Namaziandost et al. [24] intended to explore the possible interrelatedness between reflective teaching, emotion regulation and teacher immunity. To this end, they recruited 384 EFL teachers who filled electronic questionnaires for the three variables. The results revealed that the psycho-emotional constructs were significantly correlated in a way that both reflective teaching and emotion regulation were tied with immunity and altogether affected job performance of the teachers. The findings indicated that pre-service and in-service programs for language teachers need to incorporate psycho-emotional constructs in their programs to foster higher level performance of the teachers.

Moreover, Azari Noughabi et al. (2023) designed a post-induction mentoring program for in-service language teachers to develop their immunity. They initially intended to identify the dominant immunity type among Iranian language teachers. In another phase of the study, they sought to explore if development of teacher immunity was facilitated by the course they designed. In so doing, they recruited 6 teachers for an in-depth analysis and data were collected through unstructured observations and qualitative interviews. The cluster analysis of quantitative data revealed that visionary archetype and fossilized archetype were prevalent immunity types in the context of Iran. The analysis of qualitative data revealed that post-induction mentoring programs could facilitate the development of productive immunity and add new coping strategies to the teachers’ repertoire.

### 2.2 Classroom observation in EFL context

Classroom observation is traditionally believed to serve three purposes, namely professional development, reward, and promotion; however, it is widely believed by researchers and practitioners that professional development is the most effective form of classroom observation [28]. Lasagabaster and Sierra [29] state that teachers find classroom observation highly stressful and intimidating in such a way that “mere mention of observation provokes uneasiness, nervousness, and tension amongst both in-service and pre-service teachers, in the belief that their professional competence is going to be questioned or judged” (p. 450).

Classroom observations in Iranian contexts are mostly carried out in a top-down fashion in a way that policy-makers who are disengaged from actual classes provide the observers with checklists. The mention of observation provokes a feeling of uneasiness among teachers, both in-service and pre-service, as they regard observation as a judgment or questioning of their professional knowledge [30]. The uneasiness has mounted to an extent that some language school supervisors analyze teacher performance through video recordings of the classes [31]. The growing demand for learning English and the inefficiency of state school programs in developing communication skills in Iranian students have caused a dramatic increase in the number of private language schools [32].

To ensure the quality of teaching, language schools recruit supervisors to observe teachers [33,34]. In recent years a number of studies have reported the problems and opportunities that observation and supervision have raised for language teachers ([12,[35], [36], [37], [38], [39]]). Razmjoo and Rasti [38], for instance, believe that Supervisor has turned into a “notorious title” in Iran which might be a result of the dismissing power of observation that is, “teachers can be dismissed due to low performance in the observations” [12]. Azizpour and Gholami [40], on the other hand, state that even supervisors face a number of difficulties in their profession. They assert that the majority of the supervisors who are appointed as supervisors without proper education are commonly faced with ethical conflicts in firing the inefficient teachers.

Supervisory classroom observation could act as a double-edged sword in that it may help some teachers find out about their weak points, whereas other teachers might find it stressful and as [39] contend, it might trigger language teacher immunity both adaptively and maladaptively. The newly introduced concept of immunity appears to be under the influence of classroom observation. However, research addressing the relationship between classroom observation and language teacher immunity development is quite meagre.

Maladaptive immunity has been reported to lead the teachers toward callousness, pessimism, and frustration and raise the probability of teacher burnout and their eventual attrition [41]. Language teacher supervisory observation has been reported to be one of the stressors that trigger maladaptive immunity among the teachers. Therefore, in another phase of the study, the current researchers intended to explore how a professional development course with the observer himself/herself could alleviate the situation for the teachers and educate the teachers to transform their maladaptive immunity to productive immunity and enrich the teachers’ coping strategies repertoire to handle the observation process and other stressors. The cultivation of language teacher immunity, a critical yet often overlooked dimension of language teaching, holds immense significance for teacher well-being and professional success.

Maladaptive language teacher immunity, characterized by emotional detachment, negativity, and frustration, has been linked to increased susceptibility to burnout and attrition [41]. Supervisory observation, a prevalent practice in language education, emerges as a notable stressor that exacerbates maladaptive immunity in teachers. To address this pressing issue, the researchers in the current study propose a professional development course that draws on the role of the observer as a facilitator, with the overarching goal of transforming maladaptive immunity into productive immunity. This intervention seeks to equip teachers with a robust arsenal of coping strategies to effectively traverse not only supervisory observation but also the myriad of stressors that permeate the teaching profession. By fostering productive language teacher immunity, teachers cultivate resilience, enhance instructional effectiveness, and ultimately elevate their professional well-being and longevity in the field. Therefore, the present study aimed at answering the following research questions:

RQ1 Is there a significant difference between the teachers' immunity before and after the professional development course?

RQ2 How do supervisory observation and the professional development course contribute to the development of language teacher immunity among novice EFL teachers?

## 3 Method

### 3.1 Context and participants

The participants of the present study were teachers instructing kids within the age range of 7–11 and teenagers aged 12 to 16. The majority of language schools in Piranshahr, Iran where the study was carried out, use *Family and friends* [42] or *Big English* [43] for the kids and *Teen2Teen* [44], *Top Notch* [45] and *American English file* [46] for the teenagers. The language schools in this city are not very big and the number of teachers working in a language school hardly exceeds 5. The classes and the teaching process of these language schools are mostly supervised by the founders and managers of the schools.

Dobakhti et al. [47] emphasize that the prerequisite for developing a specific immunity type is having adequate years of teaching experience. Since the current study was mainly centered on the development of language teacher immunity, the researchers decided to focus on early-career teachers and novice teachers who have 0–5 years of experience in teaching English [48]. The first researcher invited 13 language teachers from different language schools, to participate in the present study. The participants were 9 female and 4 male teachers, all B.A. holders in English language and literature, English translation, and TEFL. It is worth mentioning that all the managers of the schools where the participants are working stated that they didn't observe their teachers on a regular basis and the observations were carried out occasionally. Table 1 depicts the demographic information of the participants.

**Table 1:** Demographic information of the participants.

teacher	Years of experience	Gender	Degree
T1	2	Female	B.A.
T2	1	Female	B.A.
T3	1	Female	B.A.
T4	5	Female	B.A.
T5	3	Female	B.A.
T6	3	Male	B.A.
T7	2	Female	B.A.
T8	3	Female	B.A.
T9	1	Female	B.A.
T10	4	Female	B.A.
T11	2	Female	B.A.
T12	1<	Female	B.A.
T13	2	Female	B.A.

### 3.2 Instruments

#### 3.2.1 Language teacher immunity scale

The researchers made use of Language Teacher Immunity (LTI) scale developed by [9] and consisting of 39 items with a 6-point response scale from 1 (strongly disagree) to 6 (strongly agree). The scale is composed of seven factors, i.e. teacher self-efficacy (7 items), burnout (5 items), resilience (5 items), attitudes towards teaching (5 items), openness to change (6 items), classroom affectivity (6 items), and coping (5 items).

#### 3.2.2 Narrative frame

To get a deeper understanding of the teachers' lived experiences of the observation process, the researchers decided to utilize narrative frames which targeted the supervisory classroom observation and its contribution to the teachers' language teacher immunity development. Narrative frames guide the teachers to share a story that is based on their lived experience. The teachers are asked to fill out incomplete sentences that focus on the actual experience of the teachers in the presence of the supervisors and the negative or positive impact of that presence and its contribution to their immunity development (see appendix 1). Hiver [9] states that formation of narratives plays a key role in integrating language teacher immunity into the teachers’ complex professional identities. Hiver and Dörnyei [10] are of the view that there is growing recognition that our perceptions and experiences are structured in the form of narratives and we internalize these life stories selectively in a way that we choose those narratives that allow us to a “stable core sense of identity that will in turn determine our well-being and purpose, and shape our behavior” (p. 410).

#### 3.2.3 Semi-structured interview

Many studies on language teacher immunity have recruited semi-structured interviews for their data collection [9,13,23,49]. To explore how the teachers viewed their immunity levels, the impact of the observations on their emotion, self-efficacy, and burnout, and the impact of observer's mentoring through PD course on their immunities, semi-structured interviews were run. The interview questions were inspired by the language teacher immunity scale designed by Hiver and Dörnyei [10]. The interview sessions were conducted in a matter of 30 min on average, with the longest interview taking 37 min and the shortest lasting for 24 min. The interview protocol comprised 10 questions (see appendix 2).

### 3.3 Design and procedure

The present study used mixed methods research design, more specifically explanatory sequential design. Due to the complexity of the phenomenon under investigation and in an attempt to come up with more robust and generalizable findings, the researchers triangulated the quantitative data with interviews and narrative frames. The managers of the language schools were approached to get their consent and they were asked to talk to the teachers who agreed to participate in the study. It's worth noting that this study was approved by the Ethics Committee of Urmia University, and informed consent was also obtained from the participants. The teachers were initially observed at the beginning of the semester, following which the language teacher immunity scale was administered. Then, the teachers were added to a Telegram group with the observer, i.e. the first researcher of the study. A professional development course which included 8 1-h sessions was designed to be presented by the first researcher (The details of the professional development course are discussed below). Narrative frames, designed by the researchers in light of previous studies, were distributed among the teachers to fill in the course of the professional development. The narrative frames were meant to help the teachers record important changes in their narratives of the observation process. At the end of the PD course, the teachers were invited to answer the language teacher immunity scale for the second time to find out whether the professional development course led to any significant change in their immunities. Finally, the teachers were requested to take part in semi-structured interviews to see how the PD course affected their immunities.

#### 3.3.1 The professional development course

Hiver [8] stated that the process of the development of immunity leads to maladaptive immunity formation among language teachers. This statement accentuates the importance of professional development courses. Haseli Songhori et al. [23] reported that the results of interventions in their study were very promising and fruitful in the immunity development of their participants. The professional development course utilized in the current study was designed based on the findings of the first phase of the study regarding the difficulties and threats of observation to language teachers' immunity status [39]. The first researcher who acted as the observer of the teachers' classes was assigned to present the eight-session course to the participants. As the teachers were in their early stages of teaching, it was the first time that they participated in such a PD course. The eight sessions were conducted during eight weeks, one session per week. The first two sessions were devoted to knowing the teachers' opinions about teacher observation and listening to the teachers' voices and concerns. In sessions 3–8 the mentor focused on providing information about different types of observation, observer expectations and goals, as well as coping strategies to deal with stressful situations. The mentor narrated his own experience of being observed in his early stages of teaching constantly to give a feeling of convergence to the teachers. The telegram group was used as a venue where the teachers were asked to feel free to pose questions during the process and discuss issues about the observations and the course. Another factor that was emphasized was that the observer didn't expect loyal commitment to any teaching method. The PD course also made it clear that the observer merited creativity. Needless to say, during the course, the presenter of the PD course intended to create an atmosphere free of stress for the teachers. Many other studies [13,23] have emphasized the role of stress as an important factor in maladaptive immunity development of the teachers.

### 3.4 Data analysis

The Shapiro-wilk test verified that the quantitative data were normally distributed. Therefore, a paired-sample *t*-test was utilized to identify the differences in the teachers' immunity status due to the intervention program. In analyzing the qualitative data, the researchers utilized the principles of thematic analysis [50]. Following the guidelines of thematic analysis provided the researchers with the possibility to create themes and codes inductively and analyze the data through these themes. The narrative frames were in English and the interviews were also translated into English to be more convenient. The guiding principle in the analysis focused on continuously comparing data from the questionnaires, narrative frames, and interviews to develop more precise and accurate codes [51]. Reading each teacher's interviews, the researchers extracted codes that were considered relevant to the process of immunity development of the teachers. Then, the researchers strove to develop the overarching themes in the data by cross-case analysis of the emerging codes from each participant and the secondary themes were discarded (see Table 1).

## 4 Results

### 4.1 Findings obtained for the first research question

The first research question in the current study sought to explore the difference between the teachers’ immunity before and after the professional development course. In dealing with this research question, the researchers initially analyzed the normality of the data obtained for teacher immunity prior and successive to treatment (see Table 2).

**Table 2:** Test of normality run on teacher immunity scores.

	Kolmogorov-Smirnov^a^			Shapiro-Wilk		
	Statistic	df	Sig.	Statistic	df	Sig.
Teacher Immunity	.196	13	.184	.882	13	.077

As is seen in Table 2, teacher immunity scores met the normality conditions, and hence parametric statistics (paired samples *t*-test) was run to analyze the differences between pre-treatment and post-treatment immunity scores (see Table 3).

**Table 3:** Paired samples test for teacher immunity scores.

		Paired Differences					t	df	Sig. (2-tailed)
		Mean	Std. Deviation	Std. Error Mean	95 % Confidence Interval of the Difference				
					Lower	Upper			
Pair 1	Immunity pre-treatment – Immunity post-treatment	−.24967	.14579	.04044	−.33777	−.16156	−6.174	12	.000
Pair 2	Self-efficacy – Self-efficacy post-treatment	−.31584	.39611	.10986	−.55521	−.07648	−2.875	12	.014
Pair 3	burnout – burnout post-treatment	.07155	.22620	.06274	−.06514	.20824	1.141	12	.276
Pair 4	resilience – resilience post-treatment	−.50010	.35029	.09715	−.71178	−.28842	−5.147	12	.000
Pair 5	Attitudes towards teaching – attitudes towards teaching post-treatment	−.07709	.45104	.12510	−.34965	.19546	−.616	12	.549
Pair 6	Openness to change – openness to change post-treatment	−.47900	.27435	.07609	−.64478	−.31321	−6.295	12	.000
Pair 7	Classroom affectivity – classroom affectivity post-treatment	−.08317	.20651	.05728	−.20797	.04162	−1.452	12	.172
Pair 8	Coping strategies – coping strategies post-treatment	−.36401	.28775	.07981	−.53790	−.19013	−4.561	12	.001

The results reported in Table 3 indicate that there is a significant difference between the immunity of the teachers before and after the treatment. More specifically, the professional development course is found to be influential in improving the teachers’ self-efficacy, resilience, openness to change, and coping strategies. Table 4 shows the effect sizes obtained for the immunity scores prior and successive to treatment.

**Table 4:** Paired samples effect sizes for teacher immunity scores.

			Standardizer^a^	Point Estimate	95 % Confidence Interval	
					Lower	Upper
Pair 1	immunitypretreatment - immunityposttreatment	Cohen's d	.14579	−1.712	−2.567	−.831
		Hedges' correction	.15056	−1.658	−2.486	−.804
Pair 2	selfefficacy - efficacypost	Cohen's d	.39611	−.797	−1.414	−.157
		Hedges' correction	.40905	−.772	−1.369	−.152
Pair 3	burnout - burnoutpost	Cohen's d	.22620	.316	−.248	.868
		Hedges' correction	.23359	.306	−.240	.840
Pair 4	resilience - resiliencepost	Cohen's d	.35029	−1.428	−2.197	−.630
		Hedges' correction	.36174	−1.382	−2.128	−.610
Pair 5	attitudes - attitudespost	Cohen's d	.45104	−.171	−.715	.380
		Hedges' correction	.46577	−.166	−.693	.368
Pair 6	openness - opennesspost	Cohen's d	.27435	−1.746	−2.611	−.854
		Hedges' correction	.28331	−1.691	−2.528	−.827
Pair 7	classroomaffectivity - classroonaffectivitypost	Cohen's d	.20651	−.403	−.962	.171
		Hedges' correction	.21326	−.390	−.931	.166
Pair 8	coping - copingpost	Cohen's d	.28775	−1.265	−1.990	−.512
		Hedges' correction	.29715	−1.225	−1.927	−.496

Table 4 provides further evidence as to the significant difference between the immunity of the teachers before and after the treatment. As is seen in the table, the cohen's *d* indicates a fairly large effect size, for different pairs analyzed. In what follows, the researchers present the results of qualitative analysis, and by way of doing so make an attempt to provide a detailed image of the scope and depth of the influence of the professional development course on the overall immunity of the teachers and provide excerpts of different subcategories being touched by the PD course.

### 4.2 Findings obtained for the second research question

The second research question investigated the way supervisory observation and the PD course contribute to the development of language teacher immunity among novice EFL teachers. Analyzing the data obtained through interview and narrative frames, the researchers came up with a number of dominant themes which are listed below.

#### 4.2.1 The triggering impact of supervisory observation

The analysis of the qualitative data which was conducted in light of the principles of thematic analysis, suggested by Braun and Clarke (2008), revealed that all the teachers found supervisory observation to be stressful. In what follows, the researchers primarily discuss how supervisory observation triggered language teacher immunity among the participants. One of the most recurrent themes in the interviews was *lack of confidence at the presence of the observer.* The participants, who were novice teachers, considered the supervisor a superior figure in terms of general English proficiency and the art of teaching. Therefore, they thought that their teaching would not rise to the expectations of the observer and that their teaching would even seem ridiculous. Extracts 1 and 2 illustrate this concern among the participants.

Extract 1

*When I know that the observer has been in this profession for such a long time and he has got a PhD in the area, I* feel *intimidated to teach in his presence. What would he think about me if I make a thoughtless grammatical or pronunciation mistake?!* (T3)

Extract 2

*As a* novice *teacher, I know that I have a lot to learn. I try a lot but it’s no match to the knowledge and expertise of our supervisor. I was really stressed the session he observed my class*. (T12)

The second theme was *cynicism of the teachers towards the observations.* A number of the teachers thought that the observation didn't lead to the development of the teachers, but was rather a way of identifying incompetent teachers to stop collaboration with them. In other words, they found observation an actual threat to their job and as a result, it became stressful to them. This theme is reflected in the extract below.

Extract 3

*To me, observation means lack of trust in the teacher and his/her abilities. If you trust someone, you don’t need to* constantly *check if they are doing their job with honesty or not.* (T6)

The third and the last theme in this part was *disruption of the normal order of the class*. The normal order in this context refers to the manner in which the teacher typically taught, but changed his/her way of teaching due to the observer's presence (for instance, while limiting the use of humor owing to the feeling of shyness or embarrassment). This could alternatively even impact students' cooperation and involvement in interactions in the class. The teachers thought that the observer would interpret the students' reluctance as lack of learning for which he/she would find the teacher responsible. Extracts 4 and 5 are quintessential examples referring to this theme.

Extract 4

*It was in the middle of the second term of my teaching when my class was observed for the first time. I had a very good relationship with the students and the class was very active. When the observer came to the class, suddenly all the students became silent. I felt very bad because I knew that the observer thought that they hadn’t learned much from me.* (T7, narrative data)

Extract 5

*I* can’t *tell a joke to cheer the students. When the class is so stern and formal, the students hardly pay attention to you.* (T13)

#### 4.2.2 The approachability of the observer and power relations between the observer and the teacher

One of the most recurring themes was the approachability of the observers. During the PD course the teachers felt more relieved about the observations in a way that they were assured that the observations were meant to facilitate the development of the teachers. Due to the dismissive power of the observation, sometimes the observers are dehumanized in the teachers’ eyes and are seen as evil. The professional development course provided an opportunity for the teachers to be in touch with their observers and receive feedback from them, learn how to deal with perturbances, and refer to them when they had questions or needed help via telegram application. It is a strong step towards expanding the repertoire of coping strategies by the novice teachers to develop their immunities adaptively. When the teachers are left alone after the observations, they are given the chance to ruminate the experience and grow quite indifferent. This theme is reflected in the following extract.

Extract 6

I fee*l safer to have some professional to refer to when I have a problem. It happens for everyone to face situations that you can’t control or you are not experienced enough to manage. When you feel that the observer is not there to judge your performance or measure your proficiency but he is there to see your reactions and management and later give you feedback it gives you the impression that the observer is someone just like you. He is no demon who may spy on you to get fired.* (T1, narrative data)

The stress that the teachers experience during the observations comes, to some extent, from the inequality between the observer and the teacher. The dismissive power of supervisory teacher observations has made observers superior in the power relations. In the first phase of the study in which the current researchers intended to unveil the triggering impact of supervisory observation among language teachers, many teachers mentioned this inequality in power in their interviews. T4, for instance, believed that being observed by a teacher who is more experienced and enjoys higher education than you doesn't stir this feeling of inequality.

Extract 7

When the observer mentioned that he was teaching many classes I started to pay more honest attention to his tips *and points about the observations. It gave me the impression that there was no superior-inferior dichotomy at work here and it was only about improving our teaching.* (T4)

#### 4.2.3 Clarity of purpose and expectations of the observer

One of the factors that gave rise to teachers' stress and feeling of uneasiness in observation sessions was the perceived ambiguity of the observer's expectations. As mentioned in extract 8 the teachers didn't have enough information about what would be considered ideal in the observer's eyes. As a result, it triggered negative feelings among the teachers about their self-efficacy and successful teaching in the class.

Extract 8

One *thing that troubles me is that I have very little information about the expectations of the observer. We normally don’t receive detailed feedback from the observer and this makes me think that we are not supposed to learn from the observations.* (T4)

In the professional development course, one of the main points of instruction was a detailed picture of what the observer considered to be a successful teaching session. It was made clear that the observations wouldn't be utilized to assess the capabilities of the teachers, but they would serve to mediate the development of the teachers. The expectations of the observer were elucidated and all the teachers received person-to-person feedback from the observer during the PD course. It reassured the teachers of the developmental nature of the observations and helped them acquire new strategies to deal with the observation. Extract 9 below is indicative of this notion.

Extract 9

I used to ignore the presence of the observer in the class and had satisfied myself to accept it as one the hards*hips of the profession. The PD course changed the way I look at it now. If you want to make progress as a teacher, you need to ask professionals to observe your class and give you some tips that you would have never noticed.* (T11, narrative data)

#### 4.2.4 Coping strategies and resilience

Once the system is destabilized and the equilibrium is disrupted by the observations, the teachers adopt different narratives. There are many factors that contribute to the developmental trajectories as regards teacher immunity development. The PD course, as an attempt to contribute to the immunity development of the teachers through education, intends to broaden the vision of the teachers and help them acquire new coping strategies to make them more resilient. We observed above that the results of the quantitative data analysis showcased that resilience, as a subcategory of language teacher immunity scale, was positively influenced by the PD course more than the other areas. Extract 10 illustrates that the PD course has been influential in the linking stage of self-organization of the system. The teachers have learned coping strategies to deal with the observations, which can, in turn, help make them more resilient teachers.

Extract 10

I h*ave thought a lot about becoming an English teacher in private language schools. The benefits are not significant but it is something I love a lot and I have to get along with the difficulties. Learning how to change supervisory observation from a threat to an opportunity is something that makes me move forward in this profession more easily.* (T9, narrative data)

#### 4.2.5 Becoming a life-time learner

One of the teacher traits that was underscored in the PD course, which is also a sub-category of language teacher immunity, was openness to change. The observer emphasized that the observation and the subsequent challenges that come with it might take the teachers out of their comfort zone. T8 declared that learning how to teach is a life-long journey and it doesn't finish at the end of the teacher education programs. We can infer that this teacher has formed this new narrative in light of the PD course and it may have become a part of his professional identity.

Extract 11

The observer mentioned that it is his job to monitor the teachers' practices and attract their attention to the necessary changes they should make in their teaching. It resembles the job of the teacher in the class; we have to monito*r our students' performance and help them change many erroneous elements in their language learning journey. If I don’t welcome the observer’s comments, how can I expect my students to welcome mine.* (T8, narrative data)

This narrative seems to represent the realignment stage as the teacher seems to have developed a symbiotic relationship with the observer that allows her to take advantage of the challenge that she faced in the class. The PD course seems to have provided the opportunity for T8 to find the observations more meaningful and make peace with them.

## 5 Discussion

The present mixed methods study was an attempt to find out how supervisory classroom observation and a professional development course on the observation processes contribute to the immunity development of novice language teachers. With respect to the first research question, the immunity level of the teachers was measured based on their responses to the LTI scale before and after the treatment. The results of the quantitative section of the study revealed that the professional development course had a significant impact on the immunity level of participants. This finding is consistent with the one obtained by Azari Noughabi et al. [17] who found that post-induction mentoring contributed positively to the immunity development of the teachers. The finding is also in line with that of Dobakhti et al. [47] who concluded that “tailor-made teacher immunity education and learner-difference-based teacher education” had a significant impact on the immunity development of the teachers. This finding is also corroborated by Hiver's [8,9] image of the developmental trajectories of immunity which necessitates the presence of 1) triggering dissonance, 2) providing teacher with transformative coping strategies, and 3) teacher's creation of new narratives to stabilize the newly perceived immunity.

Self-efficacy was one of the subcategories that significantly changed as a result of intervention. The possible explanation for this outcome is that when the expectations of the observer are clarified and many strategies are introduced by the observer to manage difficult situations in the class, the teachers feel relieved from stress and gain more self-confidence to become the best version of themselves. Zonoubi et al. [52] found out that self-efficacy beliefs of a group of teachers were positively developed due to an intervention program. This finding is also supported by Gholaminejad [12] who regarded ambiguity of the purpose of observation to be stressful to the teachers and a source of negative attitude towards observation.

As mentioned above, the language schools where the study participants were involved were not so renowned and no specific language teaching methods were strictly followed. The observer himself (the first researcher in the current study) who also teaches in one of these language schools has witnessed that there is no hierarchical relationship between the teachers and the observer in his workplace. Thus, the current researchers are of the view that this might have allowed the teachers to accept and internalize the contents of the professional development course more easily as they perceived them to be more down-to-earth and designed for their improvement.

Regarding the second research question, the qualitative findings revealed that the professional development course could facilitate the adaptive formation of new narratives about the observations and the supervisor himself. The character and the stance of the observer are so important that they could powerfully contribute to the teachers' interpretations about the developmental nature of in-service education. The present study revealed that the approachability of the observer and the balance in power relations could impact the teachers' immunity. The teachers displayed traces of openness to change when the recommendations for change and the introduction of new strategies were presented by an observer with whom they had a productive and positive relationship. The results indicate that through in-service programs with the observer, the teachers would be encouraged to share their concerns about the threats of observation to their professional identity; and that an approachable observer would affect their resilience, boost their coping strategies, hearten them to recruit new methods, techniques and technologies, and help them trust their own capabilities. In this regard, evidence can be provided for the current finding based on Gooran et al.’s [49] claim that the intervention programs should not focus solely on the professional content knowledge of the teachers. Teaching the teachers how to reflect and manage the environmental stressors should be emphasized in an effective development course.

Since language teacher immunity can affect the various aspects of the teachers' cognition and classroom practice (see Ref. [9]) the development of productive immunity for novice language teachers is tremendously important. The results indicated that the supervisory teacher observation can trigger and disrupt the equilibrium of the teachers’ psychology. Providing mentoring programs by the observer himself/herself can decrease the stressing impact of the observer effect and help build a productive relationship in which the teachers look up to the observer and ask for help whenever needed. Looking through the lens of self-organization, we could argue that the classroom observations and the observer act as the initial disruptive trigger that destabilize the immunity system of the teachers. However, the PD course guides the teachers through coupling and re-alignment stages towards new forms of stabilization. In other words, the PD course enables the teachers to foster adaptive teacher immunity and accompanies them through the stages of coupling and realignment by reformulating the immunity systems and providing new response options to the disruption adding to their repertoire of coping strategies [9].

## 6 Conclusion

The present study explored the role of supervisory classroom observation in the development of language teacher immunity of novice EFL teachers. The findings revealed that observation triggered teacher immunity and disrupted the equilibrium of the teachers. The results also revealed that the professional development course contributed significantly to the immunity development of the teachers. Like other studies, the current investigation is not void of limitations, among which mention can be made of the fairly small sample size utilized in the study. The future researchers might choose to get around the issue by selecting a larger sample. Furthermore, the sample used in the current study is not representative enough of all the language schools across different regions of Iran. Hence, future researchers who might want to replicate the study, are recommended to opt for a more representative sample by selecting the participants from different cities and provinces across Iran to augment the generalizability of the findings.

The implication of the findings can be that novice EFL teachers are intimidated by classroom observation and it may lead to maladaptive teacher immunity among them. To prevent that from happening, language schools need to familiarize the teachers with observations, and provide them with in-service mentoring programs. The findings indicated that the teachers improved significantly in their resilience, self-efficacy, coping, and openness to change. It means that being in touch with the observer helped the teachers not only overcome their fear of being observed, but also gain an opportunity to learn from a more experienced professional. It is completely practical for small language schools to hold mentoring programs in which the teachers socialize with their observer and find the whole process of observation less scary. The language schools need to encourage the creativity of the teachers rather than requiring them adhere to specific methodology and penalizing the teachers for deviating from that which is the case in some language schools in Iran. In sum, the results indicate that receiving proper training from the observers can be fruitful for novice EFL teachers to help improve their immunity in the face of hardships.

## CRediT authorship contribution statement

**Mohammad Sheikhi:** Writing – original draft, Project administration, Methodology, Investigation. **Parviz Alavinia:** Writing – review & editing, Validation, Supervision, Conceptualization.

## Informed consent

Informed consent was obtained from all individual participants included in the study.

## Data availability statement

It must be noted that the data associated with this article is confidential as some of the participants involved in the study didn't reveal their consent as regards making their responses publicly available.

## Ethical approval

All procedures performed in studies involving human participants were in accordance with the ethical standards of the institutional and/or national research committee and with the 1964 Helsinki declaration and its later amendments or comparable ethical standards.

## Funding

This study was not funded by any organizations.

## Declaration of competing interest

The authors declare that they have no known competing financial interests or personal relationships that could have appeared to influence the work reported in this paper.

## Footnotes

Appendix A Supplementary data to this article can be found online at https://doi.org/10.1016/j.heliyon.2024.e39355.
