# Supplementary material for: The enhancement of teacher immunity in a professional development course: Contributions from supervisory observation and the observer
Source: Heliyon. 2024 Oct 21;10(21):e39355. doi: 10.1016/j.heliyon.2024.e39355 (PMC11566852; doi:10.1016/j.heliyon.2024.e39355)
Supplement: Multimedia component 1 [file mmc1.docx]

**Appendix 1 (Narrative Frame)**

One of my most-remembered experiences of my class being observed was when ………………..

……………………………………………………………………………………………………………………………………………………………………………………………………………………………………..…………..This experience made me think (about myself, my efficacy, my emotions,etc.)……………………………………………………………………………………………………………………………………………I mention this particular experience because (effects) ………………………………………………………………………………………..…. .………………………………………………………………………………………………………………………………………. The professional development course (has changed/ hasn’t changed) my feelings about observation (please explain why, and how) …………………………………………………………………… ……………… ………… ……………………………………………………………………… …… … …… ……….. .

…………………………………………………………………… The most important thing I learned from the PD course is that ………………………………………………… ……… ………… ………………………………………………………………………………………… . ………………………………………………………………………………………………………

It is important because it helped me ………………………………………………… ……….. ……………………………………… …………………………… …………… ……………..

**Appendix 2 (Semi-structured Interview)**

1) Do you describe yourself as a successful teacher or not? 2) What is your most important memory of a big problem in the classroom while you were teaching? 3) What is your feeling about being observed? and has it been affected by the PD course you attended or not? 4) How does the behavior and approachability of the observer affect your attitude toward observation? 5) Did the PD course affect your teaching self-efficacy? 6) Did the PD course affect your resilience and the ability to perform optimally in spite of difficulties? 7) Did the PD course affect your attitude toward teaching (the way you enjoy the job or you identify yourself with it) 8) Did the PD course affect the repertoire of your coping strategies? 9) Did the PD course affect your openness to change? 10) Did the PD course affect your teaching affectivity (overall emotions, good or bad, that you experience in the classroom as a result of teaching)?
